# Kawasaki disease following SARS-CoV-2 infection: Stronger inflammation with no increase in cardiac complications

**DOI:** 10.3389/fped.2022.1036306

**Published:** 2022-11-17

**Authors:** Da Eun Roh, Young Tae Lim, Jung Eun Kwon, Yeo Hyang Kim

**Affiliations:** ^1^ Department of Pediatrics, Busan Paik Hospital, Inje University College of Medicine, Busan, South Korea; ^2^ Department of Pediatrics, School of Medicine, Kyungpook National University, Daegu, South Korea; ^3^ Division of Pediatric Cardiology, Kyungpook National University Children's Hospital, Daegu, South Korea

**Keywords:** SARS-CoV-2, COVID-19, mucocutaneous lymph node syndrome, pediatric multisystem inflammatory disease, COVID-19 related, macrophage activation syndrome

## Abstract

**Background:**

Herein we investigate the difference between Kawasaki disease (KD) with and without a recent history of SARS-CoV-2 infection.

**Methods:**

We compared the clinical characteristics of patients with KD during the SARS-CoV-2 pandemic in a single children's hospital in Korea. Fifty-two patients were enrolled and divided into group 1 (with a history of COVID-19, *n* = 26) and group 2 (without a history of COVID-19, *n* = 26) according to whether or not they contracted COVID-19 within the 8 weeks before hospitalization. Data, including clinical features and laboratory results, were analyzed and compared between groups.

**Results:**

The median age of patients was significantly higher in group 1 than in group 2 (53 months [IQR, 24–81] vs. 15 months [IQR, 6–33], *p *= 0.001). The incidence of cervical lymphadenopathy was significantly higher (*p *= 0.017), while that of BCGitis was significantly lower in group 1 (*p *= 0.023), and patients had a significantly longer hospital stay (5 days [IQR, 3–8] vs. 3 days [IQR, 3–4], *p *= 0.008). In group 1, platelet count was significantly lower (*p *= 0.006), and hemoglobin and ferritin levels were significantly higher (*p *= 0.013 and *p *= 0.001, respectively) on the first admission day. Following treatment with intravenous immunoglobulin (IVIG), the platelet count was significantly lower (*p *= 0.015), and the percentage of neutrophils and neutrophil-to-lymphocyte ratio were significantly higher in group 1 (*p *= 0.037 and *p *= 0.012). Although there was no statistical difference, patients requiring infliximab treatment due to prolonged fever was only in group 1. The incidence of cardiovascular complications did not differ between the groups.

**Conclusions:**

Post-COVID KD showed a stronger inflammatory response than KD-alone, with no differences in cardiac complications.

## Introduction

Recent evidence suggests that young children are at a higher risk of contracting coronavirus disease 2019 (COVID-19), caused by severe acute respiratory syndrome coronavirus 2 (SARS-CoV-2) than initially expected. In the United States, children and adolescents aged < 18 years accounted for 9% of all COVID-19 cases in 2020, rising to 18.4% by August 2022 (1). In Korea, the number of infected children and adolescents started to rapidly increase from January 2020, with outbreak peaks in January and April 2021 (2, 3). As of August 23, 2022, there were 5,423,799 infected children and adolescents in Korea, accounting for 25% of all confirmed cases (4).

At the beginning of the COVID-19 pandemic, it was reported that children infected with SARS-CoV-2 had mild manifestations and a favorable prognosis. However, in April 2020, hyperinflammatory shock with clinical features similar to those of Kawasaki disease (KD) was reported in children and adolescents in England (5). Since then, similar cases have been reported across Europe and the United States (6, 7). The World Health Organization (WHO) and U.S. Centers for Disease Control and Prevention (CDC) have named this new syndrome a multisystem inflammatory disease in children (MIS-C) (8–10).

The clinical manifestations of MIS-C overlap with those of KD, including fever, skin rashes, conjunctivitis, and mucocutaneous manifestations. However, MIS-C is more commonly associated with left ventricular dysfunction (30%–40%) and shock, gastrointestinal abnormalities, and neurological manifestations than KD (11). In laboratory tests, patients with MIS-C showed marked thrombocytopenia, decreased absolute lymphocyte count, and increased C-reactive protein level compared to KD patients. The age distribution also differed, with MIS-C more common in older children.

The distribution of MIS-C overlaps with the geographic distribution of COVID-19; however, the incidence of MIS-C shows distinct differences, with a lower incidence reported in Asian populations than in African American, African, Caucasian, and European populations (7, 12). In East Asian countries, including Korea and Japan, the incidence of KD is higher, while the incidence of MIS-C is lower than that in Europe and the United States, suggesting that several factors are involved in the pathogenesis of the two diseases. For this reason, it is important to identify changes in the clinical manifestations of KD or other KD-like illnesses in the COVID-19 pandemic era in Asian children with characteristics different from those of MIS-C.

In Japan, where the prevalence of KD is high, the incidence of KD or KD-like diseases during the COVID-19 pandemic is not significantly different from that before the COVID-19 pandemic, showing a tendency to decrease (13, 14). Although the prevalence of KD is higher than that in Europe and the United States, there have been relatively few reports of MIS-C in Japan (15–19).

In Korea, since the first case report of a SARS-CoV-2 infection in February 2020 (3), the number of infected children has steadily increased (20). In February 2022, the number of COVID-19 cases among children and adolescents began to increase rapidly. Several reports have focused on KD during the COVID-19 pandemic in Korea, but most were conducted in the early stage of the pandemic, from 2019 to 2020 (2, 21). Recent studies reflecting changes due to the prolonged COVID-19 pandemic or the outbreak of new variant strains, such as Omicron, are still lacking. As the COVID-19 pandemic continues and the number of children infected with SARS-CoV-2 is increasing, it is important to study the relationship between KD and previous SARS-CoV-2 infection. This study, therefore, aimed to investigate the differences between KD patients with and without a recent history of SARS-CoV-2 infection.

## Materials and methods

### Study materials and data collection

We retrospectively reviewed the medical records of 52 patients with KD admitted to Kyungpook National University Children's Hospital in South Korea between January 2022 and June 2022. Patients were divided into group 1 (with a history of COVID-19, *n* = 26) and group 2 (no history of COVID-19, *n* = 26), according to their history of COVID-19 infection within the 8 weeks before hospitalization.

During the study period, COVID-19 infection was confirmed by real-time reverse transcription polymerase chain reaction (RT-PCR). All COVID-19 infected patients confirmed by RT-PCR were reported to the Korea Centers for Disease Control and Prevention. The individual information of COVID-19 infection can be shared with the medical staff through a mobile phone message that are delivered to infected persons at the public health center. Patients without a history of infection were confirmed to be negative by RT-PCR test before admission and retested on the third day of admission to confirm that the result was negative.

The following demographic variables were analyzed from a review of the medical records: median age, sex, fever duration, total hospitalization duration, and the number of clinical symptoms that met the principal criteria. Patients with fever for more than five days and at least four of the five principal criteria (lip redness; strawberry tongue; bilateral conjunctival injection; cervical lymphadenopathy; and changes in the extremities, such as erythema and edema of the palms and soles, and rash, including redness at BCG inoculation site) were diagnosed with complete KD (22). Incomplete KD was diagnosed when patients showed less than four of the principal criteria.

The treatment strategy was established based on reports that faster initiation of intravenous immunoglobulin (IVIG) and glucocorticoid treatment in MIS-C is associated with improved prognosis (23, 24). All patients received IVIG as the 1st line treatment, and patients who did not respond to IVIG within 24 h further received high-dose glucocorticoid (intravenous methylprednisolone 30 mg/kg/day) as the second-line treatment.

The diagnostic criteria for MIS-C were as follows: (1) children and adolescents aged ≤19 years presenting with fever ≥38.0°C for ≥24 h, laboratory evidence of inflammation (elevated erythrocyte sedimentation rate (ESR), C-reactive protein (CRP), fibrinogen, procalcitonin, D-dimer, ferritin, lactate dehydrogenase (LDH), and interleukin(IL)-6; neutrophilia; lymphopenia; hypoalbuminemia), and evidence of the involvement of two or more organs (cardiac, renal, respiratory, hematologic, gastrointestinal, dermatologic, or neurological disorders), presenting with severe manifestations requiring hospitalization; (2) exclusion of any other microbial cause of inflammation (bacterial sepsis, staphylococcal or streptococcal toxic shock syndrome, enteroviral myocarditis, etc.), and (3) evidence of current or recent SARS-CoV-2 infection (positive PCR, antibody or antigen test); or COVID-19 exposure within 4 weeks before the onset of the illness (9).

Laboratory tests were performed on the first day of admission and 24 h after the completion of IVIG treatment. The following parameters were measured: white blood cell (WBC) count, percentage of neutrophils in WBC (% neutrophils), hemoglobin, platelet counts, aspartate aminotransferase (AST), alanine aminotransferase (ALT), serum total protein, albumin, ESR, CRP, procalcitonin, N-terminal pro-brain natriuretic peptide (NT-ProBNP), ferritin, triglyceride (TG), fibrinogen, and LDH. Soluble IL-2 receptor levels and natural killer (NK) cell activity were additionally tested at admission in patients with a recent history of COVID-19 infection before the diagnosis of KD.

Kobayashi risk scores were calculated for each patient to predict IVIG resistance using the following parameters: serum sodium, days of illness before the initial treatment, AST, % neutrophils, CRP, age, and platelet count. Each parameter has a score of 1 or 2, and a cut-off score of ≥4 identifies patients with a high risk of IVIG resistance (25, 26).

### Echocardiography

The following were evaluated for acute cardiac complications during the acute phase: coronary artery abnormalities, defined as dilation (z-score 2.0–2.4) or aneurysm (z-score >2.5) (22), decreased ventricular systolic function if ejection fraction was <55%, or fractional shortening was <28% (27); atrioventricular valve regurgitation defined as more than trivial mitral valve regurgitation and/or more than mild tricuspid valve regurgitation and more than trivial pericardial effusion.

### Statistical analysis

Statistical analyses were performed using IBM SPSS Statistics for Windows, version 26.0 (IBM Co., Armonk, NY, USA). Continuous variables are expressed as the median and interquartile range (25th–75th percentile), and nominal variables are expressed as percentages. The Mann-Whitney test and Fisher's exact test were used to compare the two groups. The Wilcoxon signed-rank test was used to compare test results before and after treatment. Statistical significance was set at *P < 0.05*.

### Ethics statement

This study was reviewed and approved by the institutional review board of Kyungpook National University Chilgok Hospital (approval no. KNUCH 2022-06-043).

## Results

### Clinical characteristics

The demographic and clinical data of the two groups are summarized in Table 1. The median age of patients in group 1 was significantly higher than that of patients in group 2, but there was no difference in the male-to-female ratio between the two groups. There was no significant difference in the proportion of patients diagnosed with complete vs. incomplete KD. However, in group 1. the incidence of cervical lymphadenopathy was significantly higher (*p *= 0.017) and the incidence of BCGitis was significantly lower (*p *= 0.023) than that of patients in group 2. Conjunctival injection, lip redness/fissure, strawberry tongue, skin rash, and changes in the hand and foot showed no significant differences in incidence between the groups. Only one patient in Group 1 was diagnosed with MIS-C. The patient was a 6-year-old girl with a history of COVID-19 infection within four weeks of febrile illness. She showed clinical features of complete KD, including fever for five days, conjunctivitis, redness of the lips, redness and edema of the hands and feet, skin rash, and cervical lymphadenopathy. In addition, she was diagnosed with MIS-C due to hypotension, decreased ventricular contractility, and mitral valve regurgitation on echocardiography.

**Table 1:** Comparison of the demographic and clinical characteristics between the groups.

	Group 1 (*N* = 26)	Group 2 (*N* = 26)	*p* Value
Number of patients (%)	26 (50%)	26 (50%)	
Age, months [Median (IQR)]	53 (24–81)	15 (6–33)	0.001
Male (%)	12 (46%)	17 (65%)	0.264
Clinical manifestations			
Conjunctival injection (%)	17 (65%)	22 (85%)	0.109
Erythema of lips & oral mucosa, strawberry tongue (%)	20 (77%)	21 (81%)	0.734
Erythema and edema of the hands and feet (%)	17 (65%)	15 (58%)	0.569
Cervical lymphadenopathy (%)	9 (35%)	2 (8%)	0.017
Skin rash (%)	18 (69%)	19 (73%)	0.76
Erythema and induration at BCG inoculation site (%)	6 (23%)	14 (54%)	0.023
Hypotension or shock (%)	1 (4%)	0 (0%)	1.0
Coagulopathy (%)	0 (0%)	0 (0%)	
Gastrointestinal problems (%)	0 (0%)	0 (0%)	
Incomplete KD (%)	15 (58%)	18 (69%)	0.388
Fever duration before treatment, days [Median (IQR)]	5 (4–5)	4 (3–5)	0.079
Fever duration after initiation of treatment, days [Median (IQR)]	1 (1–2.25)	1 (1–1.25)	0.455
Total duration of hospitalization, days [Median (IQR)]	5 (3–8)	3 (3–4)	0.008
Evidence of SARS-CoV-2 (RT-PCR) (%)	26 (100%)	0 (0%)	

Values are presented as medians (interquartile ranges). Abbreviations: BCG, Bacille Calmette-Guerin; KD, Kawasaki disease; SARS-CoV-2, severe acute respiratory syndrome coronavirus 2; RT-PCR, reverse transcription polymerase chain reaction.

### Laboratory findings

**Figure 1:**
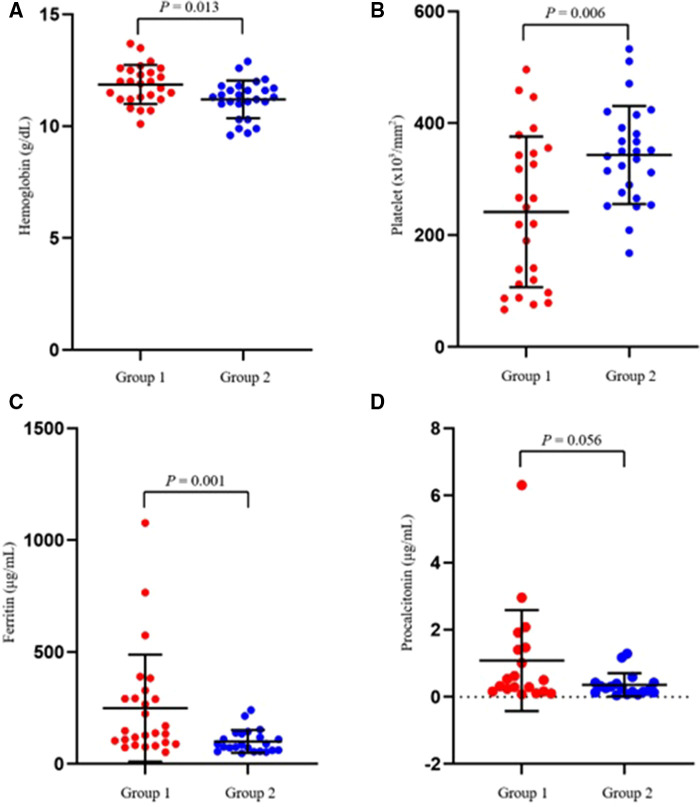
Scatter plots showing the comparative distribution of laboratory test results between the two groups. (**A**) Hemoglobin, (**B**) Platelet, (**C**) Ferritin, (**D**) Procalcitonin.

The laboratory test results of the two groups on the first day of hospitalization are summarized in Table 2. The platelet count was significantly lower (*p *= 0.006) (Figure 1), while the hemoglobin and ferritin levels were significantly higher in Group 1 (*p *= 0.013 and *p *= 0.001, respectively) on the first day of admission. The CRP, ESR, procalcitonin, albumin, AST, ALT, and NT-pro-BNP levels showed no significant differences between the groups.

**Table 2:** Comparison of laboratory findings of the two groups on the first day of admission.

Laboratory Findings	Group 1 (*N* = 26)	Group 2 (*N* = 26)	*p* Value
WBC (x 10^3^/μl)	10,615 (8,570–14,295)	13,525 (9,678–18,495)	0.111
Neutrophil (%)	71.7 (51.8–82.3)	59.1 (43.2–70.0)	0.079
Lymphocyte (%)	18.5 (10.6–35.3)	28.9 (19.5–32.9)	0.151
Monocyte (%)	5.2 (3–7.6)	5.6 (4.1–7.5)	0.332
NLR	4.0 (1.7–7.9)	2.0 (1.4–3.5)	0.103
Hemoglobin (g/L)	120 (112–125)	113 (108–117)	0.013
Platelet (x 10^3^/μl)	235 (108–349)	346 (274–398)	0.006
CRP (mg/L)	39.0 (19.9–83.0)	47.2 (34.4–72.0)	0.421
ESR (mm/h)	42 (17–60)	58 (30–74)	0.161
Procalcitonin (μg/mL)	0.52 (0.22–1.58)	0.26 (0.14–0.42)	0.056
Ferritin (μg/L)	170 (104–329)	77 (59–127)	0.001
Total protein (g/L)	63 (58–68)	62 (59–64)	0.927
Albumin (g/L)	44 (39–45)	44 (43–46)	0.276
AST (U/L)	40 (30–72)	35 (28–62)	0.197
ALT (U/L)	32 (17–93)	32 (16–80)	0.833
Total bilirubin (μmol/L)	7.9 (5.3–10.3)	7.5 (5.3–9.6)	0.978
Sodium (mmol/L)	136 (134–137)	136 (135–138)	0.933
LDH (U/L)	322 (279–362)	298 (272–328)	0.147
Triglyceride (mmol/L)	1.1 (0.8–1.4)	1 (0.8–1.5)	0.585
Fibrinogen (g/L)	4.7 (3.7–5.1)	5.1 (4.6–5.6)	0.084
NT-proBNP (pg/mL)	361 (181–1121)	482 (233–1756)	0.781

Values are presented as medians (interquartile ranges). Abbreviations: WBC, white blood cell; NLR, neutrophil-to-lymphocyte ratio; CRP, C-reactive protein; ESR, erythrocyte sedimentation rate; AST, aspartate transaminase; ALT, alanine transaminase; LDH, lactate dehydrogenase; NT-proBNP, N-terminal pro-brain natriuretic peptide.

In group 1, serum soluble IL-2 receptor and NK cell activity were measured on the 1st day of hospitalization, and the results are summarized in Table 3. NK cell activity was within the normal range, but the soluble IL-2 receptor level was above 2400 U/ml, which was set as a cutoff value for the diagnosis of hemophagocytic lymphohistiocytosis (28).

**Table 3:** Results of additional laboratory tests of group 1 on the first day of hospitalization.

Laboratory Findings	Group 1
Soluble IL-2 receptor (U/ml)	2,736 (1026–3503)
% NK cells	13.6 (4.7–13.8)

Values are presented as median (interquartile range).

Abbreviations: IVIG, intravenous immunoglobulin; IL, interleukin; NK cell, natural killer cell.

The results of laboratory tests 24 h after IVIG treatment are listed in Table 4. The platelet count was significantly lower in Group 1 (*p *= 0.015), while the percentage of neutrophils and neutrophil-to-lymphocyte ratio (NLR) were significantly higher in group 1 (*p *= 0.037 and *p *= 0.012, respectively). Changes in NLR before and after treatment were significantly higher in group 1 (*p *= 0.001). The CRP, ESR, procalcitonin, albumin, AST, ALT, and ferritin levels were not significantly different between the groups.

**Table 4:** Comparison of laboratory findings of two groups 24 h after IVIG infusion.

Laboratory Findings	Group 1	Group 2	*p* Value
WBC (x 10^3^/μl)	8,545 (6,625–12,335)	9,580 (7,492–14,532)	0.476
Neutrophil (%)	48.2 (32.5–69.7)	26.1 (17.7–43.1)	0.037
Lymphocyte (%)	42.3 (23.3–61.3)	54.7 (39.1–64.7)	0.112
Monocyte (%)	6.8 (4.8–8.1)	7.3 (6.3–8.7)	0.274
NLR	1.4 (0.7–3.1)	0.4 (0.3–0.9)	0.012
Hemoglobin (g/L)	114 (108–121)	109 (105–119)	0.24
Platelet (x 10^3^/μl)	253 (105–456)	376 (332–496)	0.015
CRP (mg/L)	21.3 (6.4–61.1)	18,1 (10.5–41.7)	0.904
ESR (mm/h)	65 (44–96)	63 (40–78)	0.672
Procalcitonin (μg/mL)	0.24 (0.06–0.7)	0.17 (0.05–0.64)	0.273
Ferritin (μg/L)	223 (158–354)	187 (149–214)	0.498
Total protein (g/L)	76 (73–83)	77 (70–80)	0.726
Albumin (g/L)	39 (36–43)	40 (38–42)	0.51
AST (U/L)	41 (31–52)	34 (30–41)	0.163
ALT (U/L)	27 (17–59)	27 (16–60)	0.856
Total bilirubin (μmol/L)	5.1 (3.6–8.2)	4.6 (3.1–8.0)	0.9
NT-proBNP (pg/mL)	804 (329–2102)	715 (271–1130)	0.427

Values are presented as medians (interquartile ranges). Abbreviations: IVIG, intravenous immunoglobulin; WBC, white blood cells; NLR, neutrophil-to-lymphocyte ratio; CRP, C-reactive protein; ESR, erythrocyte sedimentation rate; AST, aspartate transaminase; ALT, alanine transaminase; NT-proBNP, N-terminal pro-brain natriuretic peptide.

**Figure 2:**
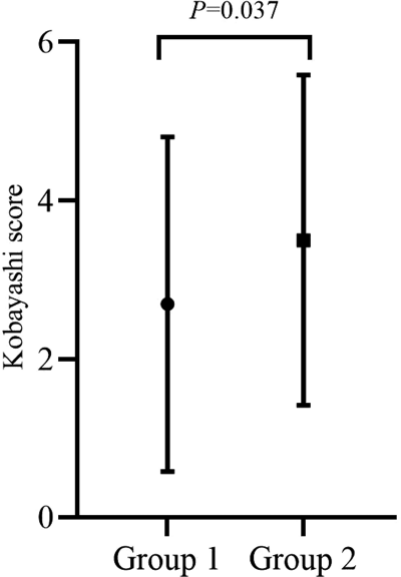
Comparison of Kobayashi scores between the two groups.

The mean Kobayashi risk score was significantly lower in group 1 (*p *= 0.037), but the median Kobayashi score was less than 4 in both groups (Figure 2).

### Clinical course

In Group 1, the total duration of hospitalization (5 days [IQR, 3–8] vs. 3 days [IQR, 3–4], *p *= 0.008) was significantly longer than that in Group 2. There was no difference between the two groups in terms of the total duration of fever before and after treatment initiation. Furthermore, there were no other differences in vasoactive/inotrope support. In group 1, the number of patients receiving intravenous methylprednisolone and infliximab was higher than group 2, but it was not statistically significant (Table 5).

**Table 5:** Comparison of medications used during acute treatment between the two groups.

Medication	Group 1 (*N* = 26)	Group 2 (*N* = 26)	*p* Value
Inotrope or vasopressor (%)	1 (4%)	0 (0%)	1.0
Intravenous immunoglobulin (%)	26 (100%)	26 (100%)	
Intravenous methylprednisolone (30 mg/kg/day) (%)	16 (62%)	10 (38%)	0.096
Infliximab (%)	2 (8%)	0 (0%)	0.49

### Cardiovascular complication

The cardiovascular complications of the two groups are summarized in Table 6. Although the patients diagnosed with MIS-C in group 1 showed decreased myocardial contractility and mitral valve regurgitation on echocardiography, the incidences of coronary artery complications, systolic dysfunction, atrioventricular valve regurgitation, and pericardial effusion did not differ between the groups. None of the patients had abnormal electrocardiogram results, such as arrhythmias or ST-segment changes.

**Table 6:** Comparison of cardiac complications in the acute phase between the two groups.

Cardiac complication	Group 1 (*n* = 26)	Group 2 (*n* = 26)	*p* Value
Coronary abnormalities			
Dilation (2.0 ≤ z score <2.5)	2 (7.7%)	2 (7.7%)	1.0
Aneurysm (z score ≥2.5)	3 (11.5%)	4 (15.4%)	1.0
Systolic dysfunction (EF < 55% or FS < 28%)	1 (4%)	0 (0%)	1.0
Atrioventricular valve regurgitation	11 (42.3%)	5 (19.2%)	0.132
Pericardial effusion	8 (30.8%)	2 (7.7%)	0.075
Arrhythmia	0 (0%)	0 (0%)	

EF, Ejection Fraction; FS, Fractional Shortening.

## Discussion

During the COVID-19 pandemic, research on the relevance of COVID-19 and KD has been conducted on various aspects. As cases of concurrent SARS-CoV-2 infection with KD have been reported (29), the possibility of SARS-CoV-2 may act as a trigger factor for KD is also being studied. The present study shows that KD following SARS-CoV-2 infection has clinically different characteristics from conventional KD. This new type of KD (Post-COVID KD) is characterized by an older age at onset and a strong inflammatory response compared to KD suspected in laboratory tests. However, the incidence of cardiac complications and clinical outcomes showed no significant difference from those of KD. Although post-COVID KD is similar to MIS-C in that it is an inflammatory response related to SARS-CoV-2 infection, it is significantly different from MIS-C in that gastrointestinal manifestations and shock are rare.

COVID-19 has spread to more than 207 countries, with 594,367,247 confirmed cases and 6,451,016 deaths worldwide as of August 23, 2022 (30). Since the first confirmed case in February 2020, there have been 22,588,640 confirmed cases and 26,224 deaths in Korea as of August 23, 2022(4). Children account for a minority of COVID-19 infections and have low severity and mortality rates in early pandemic period. However, as the household attack rate of the Delta and Omicron variants and the vaccination rate of adults has increased, the frequency of infection among children has increased accordingly.

In the present study, among the children with a history of COVID-19 infection within 8 weeks before hospitalization, only one patient was diagnosed with MIS-C. Post-COVID KD patients showed distinct clinical manifestations from those of conventional KD and did not fully meet the diagnostic criteria of MIS-C. The first case of MIS-C in Korea was reported in October 2020 (31), and as of February 2022, a total of 19 cases have been confirmed (32). In Japan, MIS-C was reported in 10 children during the Delta wave and in 8 during the Omicron wave as of March 27, 2022 (33–35). Contrary to the higher incidence of KD in Korea and Japan, the incidence of MIS-C is far lower than that in other regions, such as the United States or Europe.

MIS-C is considered a post-infectious immune-mediated disease, with a geographic distribution overlap with the area of the COVID-19 epidemic. The MIS-C epidemic curve shows a lag time of 4–5 weeks compared to that of COVID-19 (7, 36). Despite the increase in the domestic SARS-CoV-2 infection rate, there are only a few reports of MIS-C in Asian countries, possibly because the definition of MIS-C is based on the different immunological responses of Asian children and adolescents. The role of genetic factors in the pathogenesis of MIS-C, different population sizes associated with COVID-19, and pathogenic variation in SARS-CoV-2 may all be possible causes (37).

According to a nationwide survey from South Korea, KD is prevalent in children under 5 years of age, with a median age of 33 months (range: 0–205 months), and showed male predominance (22, 38). In the present study, the median age of post-COVID KD was 53 months (range: 24–81 months) showing a tendency towards an older age of onset compared to KD. The post-COVID KD group showed a lower male-to-female ratio, which was different from the results of previous studies. These epidemiologic characteristics of post-COVID KD are also different from those of MIS-C, which is reported to have an older age of onset (median age: 8–9 years), and no gender preference (7, 12, 39).

The most common symptom and sign of KD in a nationwide survey in Korea was conjunctival injection (87.8%), followed by mucosal changes, strawberry tongue, polymorphic skin rash, and changes in the hands and feet (38). In the present study, the prevalence order of signs and symptoms in Group 2 was consistent with the ranking reported in a nationwide survey in Korea. In post-COVID KD, mucosal changes were the most frequent manifestation, followed by skin rash, conjunctival infection, and changes in the hands and feet. Importantly, post-COVID KD was characterized by a higher incidence of cervical lymphadenopathy and a lower incidence of BCG site changes than KD. The significantly higher incidence of cervical lymphadenopathy and lower frequency of BCGitis in group 1 may be associated with the older median age of group 1 (40–44).

MIS-C is characterized by a high incidence of gastrointestinal manifestations and shock (4, 45), and is similar to KD shock syndrome. In post-COVID KD, gastrointestinal symptoms and decreased cardiac contractility are rare. In the acute stage of KD, leukocytosis with a predominance of neutrophils, anemia, and elevation of acute-phase reactants, such as ESR and CRP, can all occur (22, 46). Thrombocytosis is common in the first 1–2 weeks of the illness, while thrombocytopenia is rare. Lower platelet count and higher ferritin levels before IVIG treatment have been reported as risk factors for IVIG resistance in KD (38, 47). In the present study, although general inflammatory markers such as ESR and CRP did not differ between the two groups, post-COVID KD patients showed significantly lower platelet counts, higher hemoglobin and ferritin levels. The cause of these laboratory changes is unknown, but it is thought that low platelet counts and high ferritin are a transition process to a more robust immune response. NLR is also an important indicator of the balance between inflammation and immune regulation (48, 49), and an NLR exceeding 1 at 2 days after IVIG treatment was predictive of IVIG resistance and coronary artery complications in KD (50). In the present study, both groups showed a significant decrease in NLR following IVIG treatment compared with that before treatment. However, post-COVID KD patients still had a significantly higher NLR than KD patients after IVIG treatment, suggesting that post-COVID KD involved a stronger inflammatory response despite treatment.

The laboratory changes in post-COVID KD are similar to those of MIS-C in that they show neutrophilia, lymphopenia, lower platelet count, higher ferritin level, and elevated acute-phase reactants. Although MIS-C and KD had similarities in inflammatory responses, there were also important differences, such as the observance of IL-17A-mediated hyperinflammation in KD, but not MIS-C. In addition, there were differences in biomarker levels caused by differences in the T cell subset and cytokine mediators participating in the inflammatory responses (51). It is necessary to establish evidence through follow-up studies on the differences in biomarkers, such as cytokine expression, between post-COVID KD and MIS-C.

Both KD and MIS-C are associated with the development of coronary artery abnormalities, systolic dysfunction, atrioventricular valvular regurgitation, and pericardial effusions. However, the predominance of cardiac involvement differs owing to differences in the inflammatory responses of the two diseases. In KD, it was reported that arterial inflammation by IL-17A- derived plasma protein was more prevalent than in MIS-C (51, 52). In contrast, systolic dysfunction has been reported to be the most common cardiac complication of MIS-C (52–54). In the present study, only one patient in Group 1 showed systolic dysfunction on echocardiography. The incidence of coronary artery abnormalities and other cardiac complications did not significantly differ between post-COVID KD and KD despite of laboratory risk factors in post-COVID KD.

The standard treatment strategies for KD and MIS-C are similar, including IVIG and corticosteroids (22, 55, 56). In refractory cases of both KD and MIS-C, additional therapy with a monoclonal antibody against TNF-α (infliximab), monoclonal antibodies to the IL-6 receptor (tocilizumab), IL-1 receptor antagonist (anakinra), and plasma exchange have all been used (37, 57–59). The treatment guidelines for MIS-C recommend early initiation of steroids if a strong inflammatory response is expected (23). The treatment strategy used in this study is similar to the treatment guidelines for MIS-C, in that corticosteroid treatment is initiated earlier. In all patients, IVIG was used as the primary treatment, but if the fever did not resolve within 24 h after IVIG treatment or if there were signs such as shock at the time of hospitalization, high-dose methylprednisolone therapy was initiated. The number of patients requiring additional treatment at 24 h after IVIG administration was higher in post-COVID KD. In addition, while no patients received infliximab in KD without a history of COVID-19, 2 patients in KD after COVID-19 received infliximab. Although these differences were not statistically significant due to insufficient power at the small sample size, a stronger inflammation is suspected in post-COVID KD.

This single-center study analyzed the records of a small number of patients for a short study period of 6 months. As such, prospective multicenter studies are required to confirm the evidence observed in the present study. Another limitation was the inability to compare soluble IL-2 receptor and NK cell levels between groups. A significant number of patients in Group 2 did not undergo these tests. For this reason, it was difficult to determine whether there was a statistically significant difference between the groups and soluble IL-2 receptor and NK cell levels compared with the reference value of the diagnostic criteria.

## Conclusion

In conclusion, post-COVID KD was found to differ from conventional KD. We found significant differences in age and strong inflammatory reactions, including a higher incidence of cervical lymphadenopathy, a lower incidence of BCGitis, low platelet counts, higher ferritin levels, and higher NLR levels following IVIG treatment. However, there was no difference in cardiac complications. It is important to carefully identify the changes in KD during the COVID-19 pandemic.

## Data Availability

The original contributions presented in the study are included in the article/Supplementary Material, further inquiries can be directed to the corresponding author/s.
